# Notes of the Week

**Published:** 1921-09-24

**Authors:** 


### NOTES OF THE WEEK.

### A SELF-PENALISED INSTITUTION.

Each issue of Burdett's "Hospitals and Charities" contains a request from its editor for corrections of any inaccuracy, and instances are to hand that institutions which neglect to supply up-to-date information may suffer in consequence. For example, the secretary of an institution writes that "his institution is left out of consideration by all official bodies, which take as their authority Burdett's 'Hospitals and Charities.' The latest instance of our being left out is in connection with the new voluntary hospitals' committee; and this, of course, is serious." The writer proceeds to explain that his institution does not appear in "Burdett" under a particular section because, in fact, until recent years it was a different kind of institution. In consequence of the war an important development took place. News of this development never reached the editor of "Burdett," and in consequence the hospital, we are afraid through its own fault, has been placed in difficulties. That the secretary in question is responsible is clear from the fact that his plea for alteration has been postponed till now. A late correction is better than none, and will readily be adopted as soon as possible; but we cannot help remarking that he should have appreciated the importance of being up-to-date in his returns and appreciated the value of an accurate reference in the year-book before external circumstances brought the price of neglect home to him. "Hospitals and Charities" is the hospitals' official year-book, and, in the last resort, can render them complete service only if they themselves are prompt to make the most of the advantages which it confers.

### LADY ASKWITH AND THE HEALTH MINISTRY.

In a spirited letter to the *Daily Telegraph* Lady Askwith has contrasted the plight of the hospitals with the millions appropriated, but not therefore necessarily spent, on public health. In a visit to an overcrowded area in London she found a workhouse whose inhabitants had been moved to the country. The local residents hoped that the large building would be converted into flats to ease the pressure. Lady Askwith visited the place to find that it had been taken over by the Ministry of Health. "The place was crowded with officials. They had taken over the garage. They were working in the chapel. They were crowding on the top of each other." In reply to a question on the nature of their work, she learnt that it was "Insurance statistics." This leads Lady Askwith to conclude "If a sixth part of the money spent by the Ministry of Health in building and collecting statistics were made over to the hospitals to be spent by them, and accounted for, possibly, to a special committee of the House of Commons, we should see a very different state of things in a short time." No doubt we should. But Lady Askwith overlooks an objection. It is evident that these statistics do not benefit the insured person. But why should she assume that they were intended to do so? Those who have benefited by the Insurance Act are not difficult to recognise. But it has not been the compulsorily insured. It is only common-sense, therefore, that the Act was designed to benefit those who gain by this expenditure, and not those who, like the insured, are compelled to pay for it. State schemes, in the main, benefit interests. Voluntary work, in the main, benefits men and women. It is waste of time to expect a bureaucracy to reform itself. The only thing to do is to get more support for the voluntary hospitals.

### OUR WATER SUPPLY.

There is little doubt that the recently issued warnings against water-borne disease need cause no undue alarm in the Metropolis. During the prolonged drought every precaution was taken by all the London boroughs to combat the risk. The Metropolitan Water Board will assure us that London's water is satisfactorily pure in every respect and that it is filtered and examined, chemically and bacteriologically, every day. It seems that the Ministry of Health's circular is really applicable to districts where water supplies are obtained more directly from rivers and from wells. It remains a fact, however, that the recent rainfalls have still not removed the necessity for economy in the use of water, for the amount immediately absorbed by the dried-up soil was enormous.

### AN APPEAL.

The distress which would ensure if the hospitals were, through paucity of funds, compelled to close their doors is convincingly revealed in the large number of sufferers awaiting treatment at those institutions which, at this season of the year, have been temporarily closed for painting and cleaning, or through other causes. The Hospital for Women, Soho Square, where the waiting-list frequently numbers 300, reopened on August 29, and seventy patients, the full capacity of the institution, were immediately admitted. Within a week of reopening fifty-five operations were performed. The members of the Ladies' Association have assumed the entire responsibility of providing the £1,500 which, it is estimated, the projected *x*-ray apparatus will cost, and it is confidently anticipated that the apparatus will be installed before the end of the year. A personal appeal for funds by Princess Christian has met with a generous response. Any sum received in excess of the amount required for the *x*-ray apparatus will be allocated to the reduction or discharge of the mortgage debt of £1,000.

### THE COMING WINTER.

One does not dare to be pessimistic, but there are signs that the coming winter will be a very hard and difficult time for the medical and nursing staffs at hospitals and infirmaries. Already the number of patients in Poor-law infirmaries number more than they did a year ago, and there are evidences that they will mount up considerably. A great reason for this is due to the large amount of unemployment, followed by the fact that there are large numbers of families living on the border-line of starvation. The evil has spread during the out-of-work crisis to another section of the population, the better-class artisan, who, before the war, never had to go to the Poor Law for help. He has had to pocket his pride, unfortunately, and those conversant with Poor-law administration realise the new class of recipient that has sprung up during the past year. Dr. Macnamara, the Minister of Labour, realised this in an interview with a leading newspaper a few days ago. Whilst the Government were ready to provide work for the casual labourer, who could make roads and do heavy work, there could be no help for the artisan and mechanic. He would have to wait for a general improvement in trade. It is to be hoped this will come speedily and relieve the community of having to bear the cost of keeping these highly skilled men out of work. Unless they do get relief of a substantial character there is sure to be a great increase of sickness amongst them, especially with their young children.

### WALSALL GENERAL HOSPITAL.

On Monday, September 12, a disastrous fire broke out at the Hospital in the dressing store at the basement of the building. The night nurse nearest the scene of the outbreak noticed nothing unusual when going to her meal at 2.10 a.m., but at 2.30 a.m. smoke and flames were pouring up the staircase. The night nurses on duty acted with admirable promptitude, the matron, house surgeons, and lodge porter were called, the fire brigade was telephoned for and the hose pipes were coupled up and run out in a few minutes. The heat was intense, and the smoke and gases of combustion made breathing very difficult. In spite of this perfect discipline was maintained, and before the fire brigade arrived the fire was under control. The lodge porter, W. Griffiths, showed great courage in rescuing several of the maids who were cut off in their dormitory. Two of them succeeded in climbing on the roof, but were safely removed from this perilous position. The damage is extensive, a large quantity of dressings have been destroyed, and the paint scorched 80 feet from the scene of the outbreak. Too much praise cannot be given for the excellent coolness and resource shown by the nursing staff in an emergency which might well have had even more serious consequences.

### THE EFFECT OF CLOSING BEDS.

The closing of fifty beds at the General Hospital, Birmingham, is causing difficulty to the Faculty of Medicine of the Birmingham University, and the large influx of students since the war has considerably accentuated the trouble. Whilst there are many conditions peculiar to the city, which render impossible the success of the schemes adopted in London and elsewhere, the League, by house-to-house appeals organised by the different branches, is very actively engaged in raising funds for the re-opening of some of the beds. One part of the city has expressed a desire to become responsible for the maintenance of one ward, and is asking the Board of the hospital to reopen the beds forthwith. With the prospect of a substantial grant from the Hospital Saturday Fund, who have had a very good year's work, there seems good reason that the Board may be justified in reopening the closed wards. That such a happy event may take place is rendered all the more desirable by the fact that there are about 1,000 patients awaiting admission to the several hospitals throughout the city.

### A NOVEL CAMPAIGN.

West Bromwich and district are organising a campaign for the local hospital: a concentrated house-to-house collection is being made during this month, and a novelty has been introduced in the way of a competition. Competitors are invited to send in to the Campaign Committee estimates of the result in hard cash of the fortnight's house-to-house collection. Each attempt must be accompanied by a postal-order for 2s. 6d. As the nearest estimate wins a motor-car it is expected that a considerable sum will be raised in this way. Shopkeepers and tradesmen are being included in the scheme, and the Butchers' Association has already led the way with a contribution of over £90.

### A USEFUL WORK COMPLETED.

The work of the Birmingham Branch of the Red Cross Society in sending discharged men in need of rest and change of air to convalescent homes in North Wales is now complete. All the men on the waiting-list have been benefited, the funds have been spent, and the honorary organising secretary of the branch, Mr. E. C. Thomas, has resigned. The scheme began in 1917, when Mr. Thomas was approached about it. The chairman, Mr. Arthur Lane, and his committee gave active support, and three men were sent to Aberystwyth as an experiment. This was so successful that eventually nine homes were established, which provided an after-cure holiday for 1,250 men. Each home, which was personally supervised from headquarters, possessed an honorary representative and an honorary medical officer, and was also periodically inspected. Too much thanks cannot be given to the above honorary workers and to the local residents, who made the scheme so successful. To Dr. D. Ellis and Major J. L. Mathies particular gratitude is felt. The Birmingham Professions and General Trades Fund made generous grants to keep the work going.

### ENDOWED BEDS AND HOSPITAL VISITORS.

A bed has been endowed in the Llanbradach Ward of King Edward VII. Hospital, Cardiff, in memory of the late Alderman Edward John. His many friends, desiring to perpetuate his memory, chose this form of memorial. The bed endowed is that which the daughter of the late alderman once occupied for six weeks. When the cheque for a thousand guineas was handed over a large number of people visited the hospital and made a tour of the wards. Sir William Diamond in a brief speech of welcome thanked the subscribers, and said that such a gathering was unique at the endowment of a bed, and he hoped that everyone present would help the appeal of the Finance Committee, which is now busy circularising the public for individual subscriptions.

